# Preference heterogeneity in health valuation: a latent class analysis of the Peru EQ-5D-5L values

**DOI:** 10.1186/s12955-022-02079-6

**Published:** 2023-01-02

**Authors:** Suzana Karim, Benjamin Matthew Craig, Romina Arely Tejada, Federico Augustovski

**Affiliations:** 1 University of South Florida, 4202 E Fowler Ave, Tampa, FL 33620 USA; 2 McGill University, Montreal, Canada; 3 Institute for Clinical Effectiveness and Health Policy, Buenos Aires, Argentina

**Keywords:** Health valuation, Quality-adjusted life years (QALY), Heteroskedasticity, Scale heterogeneity, EQ-5D

## Abstract

**Background:**

Preference heterogeneity in health valuation has become a topic of greater discussion among health technology assessment agencies. To better understand heterogeneity within a national population, valuation studies may identify latent groups that place different absolute and relative importance (i.e., scale and taste parameters) on the attributes of health profiles.

**Objective:**

Using discrete choice responses from a Peruvian valuation study, we estimated EQ-5D-5L values on a quality-adjusted life-year (QALY) scale accounting for latent heterogeneity in scale and taste, as well as controlling heteroskedasticity at task level variation.

**Method:**

We conducted a series of latent class analyses, each including the 20 main effects of the EQ-5D-5L and a power function that relaxes the constant proportionality assumption (i.e., discounting) between value and lifespan. Taste class membership was conditional on respondent-specific characteristics and their experience with the composite time trade-off (cTTO) tasks. Scale class membership was conditional on behavioral characteristics such as survey duration and self-stated difficulty level in understanding tasks. Each analysis allowed the scale factor to vary by task type and completion time (i.e., heteroskedasticity).

**Results:**

The results indicated three taste classes: a quality-of-life oriented class (33.35%) that placed the highest value on levels of severity, a length-of-life oriented class (26.72%) that placed the highest value on lifespan, and a middle class (39.71%) with health attribute effects lower than the quality class and lifespan effect lower than the length-of-life oriented class. The EQ-5D-5L values ranged from − 2.11 to 0.86 (quality-of-life oriented class), from − 0.38 to 1.02 (middle class), and from 0.36 to 1.01 (length-of-life oriented class). The likelihood of being a member of the quality-of-life class was highly dependent on whether the respondent completed the cTTO tasks (p-value < 0.001), which indicated that the cTTO tasks might cause the Peru respondents to inflate the burden of health problems on a QALY scale compared to those who did not complete the cTTO tasks. The results also showed two scale classes as well as heteroskedasticity within each scale class.

**Conclusion:**

Accounting for taste and scale classes simultaneously improveds understanding of preference heterogeneity in health valuation. Future studies may confirm the differences in taste between classes in terms of the effect of quality of life and lifespan attributes. Furthermore, confirmatory evidence is needed on how behavioral variables captured within a study protocol may enhance analyses of preference heterogeneity.

## Introduction

In economic evaluation, valuing health outcomes on a quality-adjusted life-year (QALY) scale is a standard practice in cost utility analysis (CUA) to enable comparisons across different healthcare interventions. Many forms of disease-specific or generic instruments have been adapted to describe domains of health-related quality of life (HRQoL) in health valuation studies [1]. Using QALYs, health valuation studies assess the value of health profiles relative to health-related quality and length of life.

The EQ-5D-5L is a widely used preference-based instrument to measure and value people's health-related quality of life (HRQoL). Corresponding to the five dimensions of health, this descriptive system has five attributes: mobility, self-care, usual activities, pain/discomfort, and anxiety/depression. In the latest version of the EQ-VT protocol, each attribute has five levels—no problems, slight problems, moderate problems, severe problems, and extreme problems/unable [2].

According to EuroQol Valuation Technology (EQ-VT) protocol (2.0), the primary method to collect preference evidence on EQ-5D-5L profiles is the composite time trade-off (cTTO) technique [3]. The protocol also included ordinal tasks (i.e., paired comparisons) that were designed to overcome some of the limitations of the cTTO. For example, the cTTO method assumes that respondents do not discount their future health (e.g., pain today has the same effect on choice as pain ten years from now) [4]. Also, in paired comparisons, respondents need to indicate a preference with a discrete set of options that are likely to be less burdensome than cTTO questions that require a respondent to match two health profiles by trading off life years [5]. The inclusion of ordinal tasks within the EQ-VT protocol followed a significant period of piloting and testing of different approaches [3]. This paper examines the ordinal responses collected during the Peru EQ-5D-5L valuation study, specifically to explore the heterogeneity in EQ-5D-5L values on a QALY scale [6].

In the analysis of preference evidence, heteroskedasticity and heterogeneity have long been identified as potential issues that need to be further explored. Preference and behaviors vary within a sample, and different methodologies have been adapted to address those issues in analyzing and interpreting preference evidence [7]. Similarly to the presence of individual preference differences, heteroskedasticity, another source of outcome variability, refers to differences in the variance of the response variable attributable to observable (e.g., task-level) factors. It is important to point out that heteroskedasticity should not be considered a form of preference heterogeneity in case the differences in response variability are induced solely by task characteristics rather than by individual differences in utility valuations.

When latent, preference heterogeneity refers to the extent of variation of individuals' tastes caused by sources that are not observed by the researchers. The effect of these unobservable sources on preferences can be channeled through different latent pathways. First, respondents systematically like or dislike different alternatives that reflect the relative importance of the attributes, and individuals with similar relative importance can be grouped into clusters or classes. (i.e., taste classes). Thus, taste heterogeneity refers to differences in the relative effects of each attribute level. For example, some respondents may weigh improved health profiles with shorter lifespans; others may prefer living longer with compromised health. Another source of heterogeneity is scale heterogeneity, which refers to more subtle differences in the absolute effects of all attributes. Individuals with similar scales can also be grouped into a scale class. Scale class differences may be related to differences in utility caused by differences in the randomness of individual behavior that creates scale variation across persons [8]. Previously, some health valuation papers observed preference heterogeneity using the standard latent class model where no scale issues were controlled for [9, 10]. However, estimating differences in attribute importance between respondents without controlling for scale heterogeneity can often mislead the interpretation of taste heterogeneity, which is confounded by scale heterogeneity [11].

Using data on respondent characteristics and behaviors, a scale-adjusted latent class (SALC) model [12] can account for taste and scale heterogeneity simultaneously by identifying latent classes of persons who differ in their relative importance (taste classes), as well as latent scale classes – groups of people who differ by how intense (or indifferent) their preferences are. Furthermore, by accounting for task complexity, we adapt this model to allow for heteroskedasticity within each scale class. A similar fashion of the heteroskedastic SALC model has been applied by Rigby et al. [13] in Best–Worst Scaling (BWS) data and Karim et al. [14] in health valuation in pits scale. To our knowledge, no studies previously estimated the heteroskedastic SALC model on a QALY scale.

In this paper, we hypothesize that both taste and scale latent classes co-exist within a national population and that heteroskedasticity is associated with observable differences in scale at task-level characteristics. Hence, the paper aims to demonstrate the implications of controlling heteroskedasticity and heterogeneity simultaneously in health valuation and separating taste and scale heterogeneity on a QALY scale. In addition, we also hypothesized that the time preference among individuals does not hold the constant proportionality assumption, and the marginal value of lifespan follows a decreasing pattern (i.e., discounting, time preferences) [4]. The results of this secondary analysis of the Peru EQ-5D-5L study have implications for future analyses of ordinal responses in health valuation, particularly those that attempt to characterize the values of a heterogeneous population.

## Methods

### Study design and data collection

The details of the Peru EQ-5D-5L study design and data collection process can be found in the original paper [6]. In brief, the study followed version 2 of the EQ-VT protocol developed by the EuroQol Group, which was developed specifically for valuing EQ-5D-5L health states using computer-assisted personal interviews [2]. As mentioned in the original paper, a random sample of adults (N = 1000) was recruited for a household survey in Lima, Arequipa, and Iquitos. Some of the respondents (N = 300) were randomly selected to complete 11 cTTO tasks prior to the ordinal ones. All respondents completed ten paired comparisons with five EQ-5D-5L attributes (i.e., the latent scale pair A vs. B) followed by twelve matched pairs (i.e., A vs. B and B vs. C) with EQ-5D-5L and lifespan attributes. Within the matched pairs, 50% of the respondents received questions with a shared lifespan attribute in the first pair and the rest with differential lifespan attributes. An example of a paired comparison and matched pair is presented in Fig. 3.

### Econometric analysis

For the original publication, Augustovski et al. [6] estimated EQ-5D-5L values on a QALY scale for the general population of Peru. This econometric analysis extends its predecessor's logit estimation by examining heterogeneity and heteroskedasticity in health valuation.

#### Specification of health value

In this analysis, the value $(V)$ of a health outcome is defined on a QALY scale, where there holds a proportional relationship between the independent values of quality of life ${V}_{1}(Q)$ and length of life${V}_{2}(T)$.^1^ The quality of life is specified by EQ-5D-5L health profiles^2^ where we have used an additive regression (${V}_{1}\left(Q\right)=\left(1-{\beta }^{\mathrm{^{\prime}}}X\right)))$ to show the effect of quality of life on the value of health. Here, $X$ is the EQ-5D-5L attributes, and $\beta$ shows the incremental difference between severity levels under each dimension.^3^ For example, ${\beta }_{2}$ represents the difference in value between severity levels 2 and 3 under the mobility dimension [${MO}_{\mathrm{2,3}}$]. By construction, all coefficients on the incremental dummies (${\beta }_{k} ; k=1\; to\; 20)$ are hypothesized to be positive and represent losses in quality due to increases in the level of severity of a health condition from the full health profile 1. The value of the length of life, ${V}_{2}\left(T\right),$ is defined by a power function (${V}_{2}\left(T\right)= {T}^{\alpha }$) with power $(\alpha )$ less than one to capture discounting effect of future health.

$$V={V}_{1}\left(Q\right){\times V}_{2}\left(T\right)$$

$$V=\left(1-{\beta }^{^{\prime}}X\right)\times {T}^{\alpha }$$

$$V= \left(1- \left(\begin{array}{c}{\beta }_{1}{MO}_{\mathrm{1,2}}+{\beta }_{2}{MO}_{\mathrm{2,3}}+ {\beta }_{3}{MO}_{\mathrm{3,4}}+{\beta }_{4}{MO}_{\mathrm{4,5}}+\\ {\beta }_{5}{SC}_{\mathrm{1,2}}+{\beta }_{6}{SC}_{\mathrm{2,3}}+ {\beta }_{7}{SC}_{\mathrm{3,4}}+{\beta }_{8}{SC}_{\mathrm{4,5}}+\\ {\beta }_{9}{UA}_{\mathrm{1,2}}+{\beta }_{10}{UA}_{\mathrm{2,3}}+ {\beta }_{11}{UA}_{\mathrm{3,4}}+{\beta }_{12}{UA}_{\mathrm{4,5}}+\\ {\beta }_{13}{PD}_{\mathrm{1,2}}+{\beta }_{14}{PD}_{\mathrm{2,3}}+ {\beta }_{15}{PD}_{\mathrm{3,4}}+{\beta }_{16}{PD}_{\mathrm{4,5}}+\\ {\beta }_{17}{AD}_{\mathrm{1,2}}+{\beta }_{18}{AD}_{\mathrm{2,3}}+ {\beta }_{19}{AD}_{\mathrm{3,4}}+{\beta }_{20}{AD}_{\mathrm{4,5}}\end{array}\right)\right)\times {T}^{\alpha }$$

#### Specification of model

The standard framework for the analysis of ordinal response data is the random utility model (RUM). The underlying assumption of the random utility model (RUM) is that an individual considers some alternatives and chooses the alternative that results in the highest expected utility at any given choice occasion. In a simple setup, each individual is faced with a particular choice task where they present a pair of alternatives (i.e., health profiles) and are asked to choose the preferred one between the two outcomes in each pair. In health valuation, this approach to health profiles is known as the episodic random utility model, which is distinct from its instant and angular counterparts [15].

In this health valuation study, respondents consider multiple health profiles across the paired comparison tasks. For each respondent $i$, utility $({U}_{itj})$ of health profile, $j$, in the choice situation, $t$, is composed of an explainable component, ${V}_{itj}$ and an unexplainable random component ${\varepsilon }_{itj}$ (i.e., ${U}_{itj}={V}_{itj}+ {\varepsilon }_{itj}$), where ${V}_{itj}$ included both quality and quantity attributes with associated coefficients (${\beta }_{k}\; and\; \alpha ; k=1\; to \;20)$ to be estimated.

#### Conditional and heteroskedastic logit

Considering the random component of each health profile as an extreme value type 1 distribution, the choice per task formed logistic distribution, and under the conditional logit [16] framework, for individual $i$ the probability^4^ of selecting health profile $j$ in task $t$ is,

$$P\left( {y_{itj} = 1|\beta ,\alpha ,\mu } \right) = \frac{{{\text{exp}}\left( {\mu V_{itj} } \right)}}{{\mathop \sum \nolimits_{g = 1}^{J} {\text{exp}}\left( {\mu V_{itg} } \right)}} = \frac{{\exp \left( {{\upmu }\left( {\left( {1 - \beta^{{\prime }} X_{itj} } \right) \times T_{itj}^{\alpha } } \right)} \right)}}{{\mathop \sum \nolimits_{g = 1}^{J} \exp \left( {{\upmu } \left( {\left( {1 - \beta^{{\prime }} X_{itg} } \right) \times T_{itg}^{\alpha } } \right)} \right)}}$$

The conditional logit (CL) model is commonly used on ordinal tasks data for health preference studies, and the model gives an average preference weight $$(\beta )$$ to each attribute and a power ($$\alpha$$), which are homogeneous for everyone with a constant scale parameter, $$\mu$$. The parameters are estimated in a natural log scale (i.e., log odds ratio), and the scale is usually assumed to be 1. In this analysis, we measured preference in a QALY scale, and we would need the appropriate transformation procedure of the log-odds scale to the QALY scale. An important issue regarding the logit model specification is that the health value is defined by the difference in health profiles between the paired alternatives in each task. The value of 1 QALY, which is the difference between the two anchor points [immediate death (T = 0) and full health (11,111) with T = 1 year], is captured by the scale parameter $$\mu$$. Hence, rather than fixing the scale parameter, $$\mu$$, we estimated it, which worked as a conversion factor from the QALY scale to the log odds scale. In a heteroskedastic logit, the constant error variance assumption of the CL model was relaxed by assuming that its variance may vary between tasks systematically in response to task format. Variations in task format ($${Z}_{3})$$ had been hypothesized to influence the scale parameter (i.e., scale and variance are inversely related), namely, whether the task is a latent scale pair or a matched pair and the duration to answer each of the tasks. In order to have scale as a non-negative value, the scale parameter was modeled as follows:

$$\mu_{it} = {\text{exp}}\left( {\gamma^{{\prime }} z_{3it} } \right)$$

where ${z}_{3it}$ are hypothesized variables that affect the scale, and testing the sign and significance of associated $\gamma$ parameters explains how task characteristics influence the scale (i.e., heteroskedasticity).

#### Latent class analysis of taste and scale heterogeneity

Preference heterogeneity from latent sources may be modeled using individual-specific or class-specific parameters (e.g., mixed or latent class logit). The scale-adjusted latent class (SALC) analysis used in this study examined class-specific heterogeneity (scale and taste) in the presence of heteroskedasticity [8, 13, 17]. The SALC model is an advanced econometric approach where in addition to the standard decomposition of the population into $M$ distinct taste classes, the model allows for heterogeneity in scale with distinct $S$ scale classes (each with relatively different error variances). The model allows controlling for the differences in the error variance by assuming that despite sharing the same taste structure within the same class (${\beta }_{m}$ and ${\alpha }_{m}$), some people may display different levels of uncertainty (${\gamma }_{s}$), thereby belonging to different scale classes (s) [12]. Like a heteroskedastic logit model by class, the probability of choice then becomes:

$$P\left( {y_{itj} = 1{|}s,m} \right) = \frac{{\exp \left( {\exp \left( {\gamma_{s}^{{\prime }} z_{3it} } \right) \left( {\left( {1 - \beta_{m}^{{\prime }} X_{itj} } \right) \times T_{itj}^{{\alpha_{m} }} } \right)} \right)}}{{\mathop \sum \nolimits_{g = 1}^{J} \exp \left( {\exp \left( {\gamma_{s}^{{\prime }} z_{3it} } \right) \left( {\left( {1 - \beta_{m}^{{\prime }} X_{itg} } \right) \times T_{itg}^{{\alpha_{m} }} } \right)} \right)}}$$

**Fig. 1:**
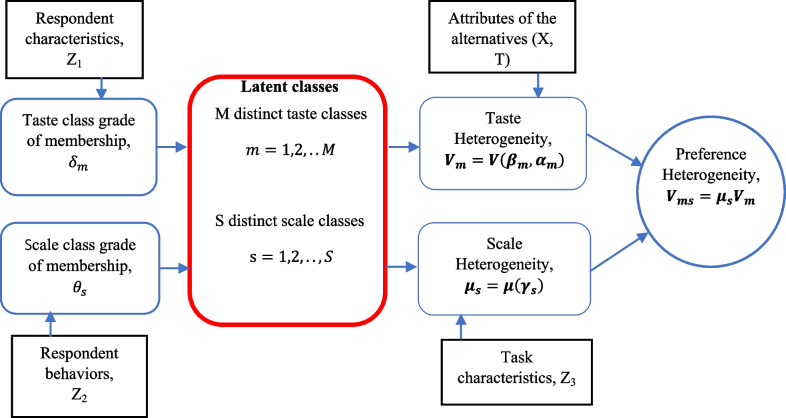
The SALC analysis in health valuation

The diagram in Fig. 1 shows the pathway of the SALC model to identify scale and taste classes separately. To avoid misidentification of taste and scale classes, we have used separate sets of covariates to identify each individual's taste and scale class membership (${Z}_{1}$ and ${Z}_{2}$, respectively) as well as the third set of covariates ${Z}_{3}$ to identify heteroskedasticity within each scale class [8].

The difference among the taste classes captured the difference in taste variation across groups of people by different attribute coefficients across classes once the scale was adjusted. So, we hypothesized that the sources associated with the likelihood of belonging to a taste class ${Z}_{1}$ include individual characteristics (e.g., demographics—age, gender) and the subject's experiences (e.g., self-stated health condition, whether they care for themselves or family members, and whether they have completed the cTTO task prior to ordinal tasks). As for the potential sources of scale heterogeneity, individuals' likelihood of belonging to a particular scale class was considered to be associated with the irregularities and idiosyncratic features of choice behavior which were not associated with any particular attribute level which captured the variability across subjects. The variables to identify the scale class membership of each individual ${Z}_{2}$ were demographic characteristics (age, gender), choice uncertainty (e.g., difficulties in understanding the questions, attribute difference, and choosing the best answer), perception (e.g., completed cTTO questions or not), and behavioral phenomena (e.g., length of the survey). All grade-of-membership variables were included as dummy variables in the model.

The taste and scale class membership probabilities are as follows:

$$p\left( {m|{\updelta }} \right) = \frac{{\exp \left( {\delta_{m}^{{\prime }} z_{{1{\text{i}}}} } \right)}}{{\mathop \sum \nolimits_{j = 1}^{M} \exp \left( {\delta_{j}^{{\prime }} z_{{1{\text{i}}}} } \right)}}$$

$$p\left( {s|{\uptheta }} \right) = \frac{{\exp \left( {\theta_{s}^{{\prime }} z_{{2{\text{i}}}} } \right)}}{{\mathop \sum \nolimits_{j = 1}^{S} \exp \left( {\theta_{j}^{{\prime }} z_{{2{\text{i}}}} } \right)}}$$

And the full choice model for each respondent $i$ becomes

$$P\left( {y_{i} {|}\beta ,\alpha ,\gamma ,\delta ,\theta } \right) = \mathop \sum \limits_{m = 1}^{M} P\left( {m{|}\delta } \right)\mathop \sum \limits_{s = 1}^{S} P\left( {s{|}\theta } \right)\mathop \prod \limits_{t = 1}^{T} \mathop \prod \limits_{{{\text{j}} = 1}}^{J} P\left( {y_{itj} |s,m} \right)^{{y_{itj} }}$$

Under a SALC framework (Fig. 1), a respondent's probability of a choice depends on the attributes of the health profiles (*X* and $T$) and the respondent's characteristics and behaviors as well as task characteristics $(Z_{1} ,Z_{2}$, and $Z_{3}$ respectively). Due to the lack of data and convergence issues, models with more than three taste classes or two scale classes were not estimated. This secondary analysis was conducted in recognition that the original study is not sufficiently large enough to identify the full breadth of taste and scale classes within the Peru general population. Statistical analyses were done in R 4.0.2 [18–20]. Following the standard practice of maximization routine for latent class models, we have conducted Maximum likelihood estimation using the expected-maximization (EM) algorithm procedure [21]. For the maximization of the expected function in each step of the algorithm, we used the maxLik package [19]. Multiple starting values using the random number generator were used to prevent ending up with a local solution of the likelihood estimation (“Appendix 2”) [21, 22]. The best-fitted model with the optimal number of taste and scale class combinations is identified using the Bayesian information criterion (BIC) [23]. A significance level of 0.05 was considered statistically significant.

## Results

In total, 1000 respondents were interviewed for the study. Augustovski et al. [6] has a discussion on the sample background characteristics in detail. This secondary analysis was based on a balanced panel (i.e., respondents completed all tasks) with 983 respondents and dropped 17 respondents who did not complete the whole questionnaire. Each respondent has 34 completed tasks [e.g., 10 latent scale pairs, 12 matched pairs (A vs. B), and 12 matched pairs (B vs. C)].

### Difference between homoskedastic and heteroskedastic results

Table 1 presents the conditional logit (CL) model and heteroskedastic logit (HCL) model results. From the CL to HCL model, the BIC value decreased 38,981.67–38,383.63, which showed that the HCL is a better fitted model. Also, the HCL model has consistent estimates with all positive coefficients in quality-of-life (QoL) attributes, whereas two of the 20 QoL coefficients in the CL model had negative estimates. Except for the changes from slight to moderate problem under usual activity and no problem to slight problem under anxiety/depression, all the other coefficients were statistically significant (p-value < 0.05) in the HCL model. The ranking of coefficients of both models showed similar order where the maximum effects in decrement of health value occurred from changes in attribute level from severe to extreme across all the dimensions. Compared to the CL, the standard error is improved by around 29.10% among the QoL attributes in the heteroskedastic model. Also, the correlation between the QoL coefficients is 0.887 (Lin's concordance; 95% CI 0.808–0.935). These results suggested that the heteroskedastic model improved the model fit with coherent coefficient estimates and improved precision level.

**Table 1:** Value set from the conditional and heteroskedastic logit model

	Conditional			Heteroskedastic		
	Coef.	S.E	p-value	Coef.	S.E	p-value
Mobility						
Level 1–2	0.029	0.013	0.032	0.075	0.010	< 0.001
Level 2–3	0.013	0.014	0.357	0.021	0.011	0.046
Level 3–4	0.171	0.014	< 0.001	0.143	0.011	< 0.001
Level 4–5	0.250	0.015	< 0.001	0.201	0.012	< 0.001
Self-care						
Level 1–2	− 0.021	0.014	0.132	0.030	0.011	0.006
Level 2–3	0.051	0.014	0.000	0.023	0.011	0.038
Level 3–4	0.113	0.014	0.000	0.104	0.011	< 0.001
Level 4–5	0.127	0.015	< 0.001	0.100	0.011	< 0.001
Usual activity						
Level 1–2	0.010	0.014	0.487	0.042	0.011	< 0.001
Level 2–3	0.005	0.014	0.747	0.010	0.011	0.367
Level 3–4	0.159	0.014	< 0.001	0.115	0.011	< 0.001
Level 4–5	0.202	0.014	< 0.001	0.175	0.011	< 0.001
Pain/discomfort						
Level 1–2	0.022	0.014	0.105	0.052	0.011	< 0.001
Level 2–3	0.032	0.014	0.021	0.027	0.011	0.012
Level 3–4	0.154	0.014	< 0.001	0.126	0.011	< 0.001
Level 4–5	0.196	0.014	< 0.001	0.144	0.011	< 0.001
Anxiety/depression						
Level 1–2	− 0.036	0.014	0.009	0.006	0.011	0.576
Level 2–3	0.099	0.014	< 0.001	0.066	0.011	< 0.001
Level 3–4	0.097	0.014	< 0.001	0.088	0.011	< 0.001
Level 4–5	0.150	0.015	< 0.001	0.116	0.011	< 0.001
Lifespan in years						
Power value	0.251	0.012	< 0.001	0.396	0.016	< 0.001

*Coef.* coefficient, *S.E* standard error

While looking at the scale function, we saw some interesting effects of task-level characteristics on the scale under the specification of the HCL (Table 4). The intercept of the heteroskedastic model corresponds to the logarithmic scale parameter for the reference category, i.e., latent pair tasks completed between 15 and 29 s. As we are estimating the coefficients on a QALY scale, in addition to the variability, the intercept also represents the conversion value from the QALY scale to the natural scale of the logit model. So, based on the results, the intercept represents the conversion of QALY to log odds as 4.020 for latent pair responses which were completed between 15 and 29 s, and matched pair responses increased variability by negatively affecting the scale, 1.077.^5^ Under the latent scale pair responses, compared to the responses that took between 15 and 29 s to complete, other response time categories (either less than 15 or greater than 29 s) were associated with lower values for the scale parameter. And under matched pair, if the task completion time was between 1 and 14 s, it positively affected the scale compared to the baseline period of 30–59 s (p-value < 0.05).

### Latent class analysis of taste and scale heterogeneity

We conducted latent class analyses with up to three taste and/or scale classes. The estimated latent class models with an unadjusted scale converged successfully up to two classes; however, in SALC analysis, the highest converged model is the ‘2scale-3taste’ class. The BIC values among the converged models showed that the model with two scale classes and three taste classes has the lowest BIC value (35,112.00) (Table 5). So, we have selected the ‘2scale-3taste’ class model as the best-fitted model to show scale and taste variation among the respondents.

Among the three taste classes in the ‘2scale-3taste’ class SALC analysis, the first taste class is considered the most quality-of-life oriented class with the largest coefficients in all health attributes and with the lowest coefficient value in the power of the lifespan attribute. Around 33 percent of the respondents fell into this class and preferred the quality of their health more than their lifespan (Table 2, column 1) in terms of valuing health. All the coefficients in this class had the expected positive sign. Across all the dimensions, the highest loss in QALY occurred at the changes in level from severe [4] to extreme [5], and the least loss in QALY occurred from changing levels from slight [2] to moderate [3]. This class was also most sensitive to pain/discomfort. The coefficients associated with changes in severity levels from slight to moderate, moderate to severe, and severe to extreme are the highest in the pain/discomfort dimension compared to other dimensions. Having the lowest power value of lifespan attribute indicated that the group of people associated with this class had the highest decreasing marginal value of lifespan with respect to lifespan. For health profiles such as 55,555, 44,444, and 33,333, the health value associated with this class was worse than death, − 2.114, − 0.958, and − 0.130, respectively. Also, the QALY range in this class was from − 2.114 to 0.866.

**Table 2:** Value sets of three taste classes in scale class one of the two-scale class three-taste class model

	Taste class 1 (33.35%) Quality-of-life oriented			Taste class 2 (39.71%) Middle			Taste class 3 (26.72%) Quantity-of-life oriented		
	Coef.	S.E	p-value	Coef.	S.E	p-value	Coef.	S.E	p-value
Mobility									
Level 1–2	0.163	0.023	0.000	0.056	0.013	< 0.001	0.004	0.010	0.701
Level 2–3	0.046	0.026	0.074	0.010	0.013	0.445	0.018	0.010	0.067
Level 3–4	0.214	0.028	0.000	0.124	0.013	< 0.001	0.050	0.010	< 0.001
Level 4–5	0.244	0.029	< 0.001	0.187	0.015	< 0.001	0.112	0.012	< 0.001
Self-care									
Level 1–2	0.160	0.025	< 0.001	0.019	0.014	0.188	− 0.004	0.011	0.703
Level 2–3	0.050	0.027	0.058	0.006	0.013	0.633	0.023	0.010	0.025
Level 3–4	0.123	0.026	< 0.001	0.096	0.013	< 0.001	0.042	0.010	0.000
Level 4–5	0.124	0.027	< 0.001	0.109	0.014	< 0.001	0.047	0.010	< 0.001
Usual activities									
Level 1–2	0.161	0.026	< 0.001	0.026	0.014	0.056	− 0.004	0.010	0.720
Level 2–3	0.049	0.026	0.057	0.008	0.013	0.525	0.000	0.010	0.966
Level 3–4	0.159	0.026	< 0.001	0.109	0.014	< 0.001	0.041	0.011	0.000
Level 4–5	0.267	0.029	< 0.001	0.167	0.015	< 0.001	0.088	0.011	< 0.001
Pain/discomfort									
Level 1–2	0.134	0.024	< 0.001	0.035	0.013	0.005	0.008	0.010	0.409
Level 2–3	0.100	0.024	< 0.001	0.023	0.012	0.063	0.010	0.010	0.325
Level 3–4	0.207	0.027	< 0.001	0.116	0.013	< 0.001	0.041	0.011	0.000
Level 4–5	0.285	0.029	< 0.001	0.109	0.015	< 0.001	0.072	0.011	< 0.001
Anxiety/depression									
Level 1–2	0.166	0.025	< 0.001	− 0.022	0.013	0.099	− 0.009	0.010	0.340
Level 2–3	0.101	0.026	0.000	0.046	0.013	0.000	0.028	0.010	0.006
Level 3–4	0.126	0.029	< 0.001	0.073	0.014	< 0.001	0.033	0.010	0.001
Level 4–5	0.236	0.029	< 0.001	0.086	0.014	< 0.001	0.045	0.011	< 0.001
Lifespan in years									
Power	0.070	0.026	0.007	0.237	0.017	< 0.0001	0.494	0.029	< 0.001

*Coef.* coefficient,* S.E* standard error

Contrary to taste class 1 (quality-of-life oriented class), taste class 3 (Table 2, column 7) had the least effect on the health attributes and the largest effect on lifespan attribute in valuing health. This means around 26.72 percent of the respondents who belonged to this class preferred to value their lifespan (e.g., the quantity of life years) more than their quality of life compared to the other two taste classes. There were negative signs in three coefficients (level change from no problem to slight problem in dimensions mobility, self-care, anxiety/depression). Even in the extreme health condition across all dimensions, 55,555, they possessed a positive value of life (0.356), and the QALY ranged between 0.356 and 1.009.

Lastly, taste class 2 in Table 2 was comprised of around 40 percent of the respondents. This class is considered a middle class whose coefficients fell between the quality-of-life oriented class (taste class 1) and length-of-life oriented class (taste class 3). For this class, the highest QALY loss occurred due to the inability to walk, followed by usual activity, pain/discomfort, self-care, and anxiety/disorder. Although at the extreme condition of 55,555, the health value is lower than death (− 0.384), in situations such as 44,444 and 33,333, the QALY is positive, 0.274, and 0.792, respectively. The power value of lifespan attributes indicated that the marginal value of life decreases faster than the quality-of-life oriented class and slower than the length-of-life oriented class. The QALY range in this class was − 0.384 to 1.022.

**Fig. 2:**
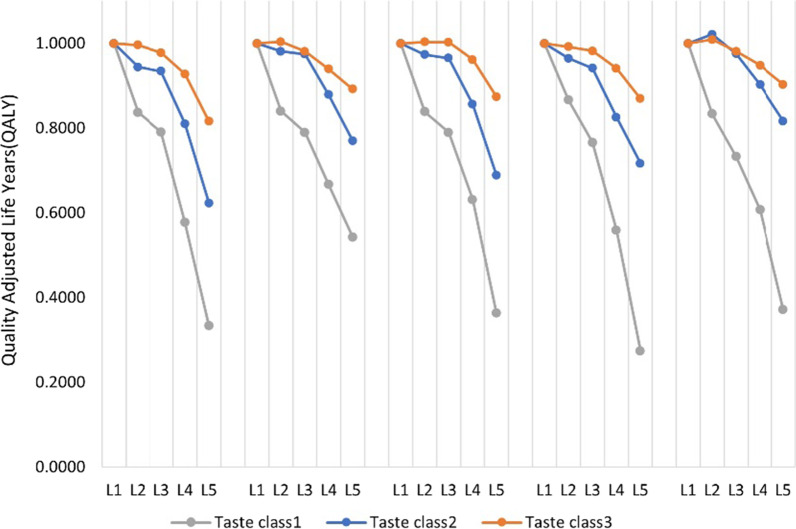
The effect of health problems on a quality-adjusted life-year

**Fig. 3:**
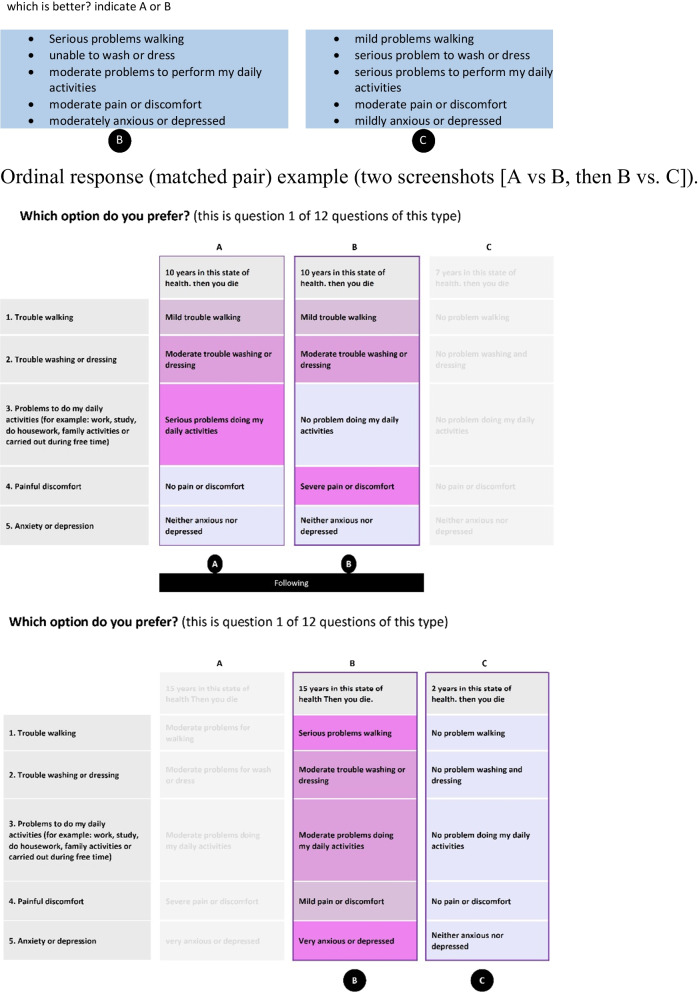
Latent-scale (single-pair ordinal response). Initial screen example

**Fig. 4:**
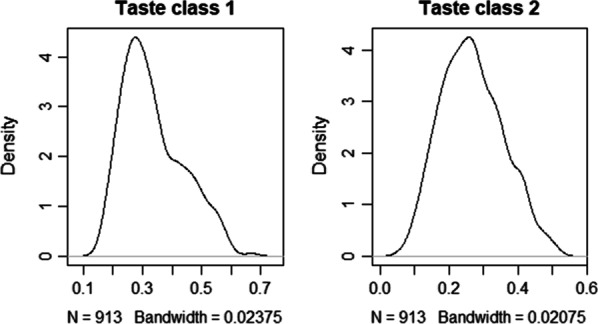
Distribution of Individual grade-of-membership of taste classes in 2scale-3taste class SALC model

The distribution of individual grade-of-membership in taste class 1 and taste class 2 from the 2scale-3taste class SALC model is shown in Fig. 4. The likelihood of being a member of the highest quality-sensitive class (taste class 1) was highly dependent on whether the respondent completed the cTTO task (Table 3). Those who completed the cTTO tasks were more likely to be in the quality-of-life oriented class (i.e., the odds ratio of the cTTO coefficient is less than 1 for taste class 2 and taste class 3 grade-of-membership), i.e., they found health problems to be relatively more burdensome. For example, the effect of being at the extreme stage in any health dimension (Fig. 2) reduces the value from full health (QALY 1) to the lowest level in taste class 1. On the other hand, the effect of not completing the cTTO task was higher in taste class 3 than in taste class 2, and for the same health detriment, the burden was higher in taste class 2 compared to taste class 3. In summary, completing the cTTO task caused the Peru respondents to inflate the burden of health problems on a QALY scale compared to those who did not complete the cTTO task (i.e., framing).

**Table 3:** Taste class grade-of-membership variables of the SALC model

	Taste class 2 (39.71%)			Taste class 3 (26.72%)		
	Coef.	S.E	p-value	Coef.	S.E	p-value
Intercept	0.996	0.382	0.991	1.240	0.608	0.660
Age						
15–29	1.480	0.655	0.376	1.000	0.275	0.999
30–44	.	.	.		.	.
45–59	1.234	0.282	0.358	1.239	0.098	0.006
60–80	0.961	0.332	0.907	0.727	0.213	0.278
Gender						
Female	0.892	0.228	0.656	− 0.326	0.194	0.226
Self-rated health using EQ VAS						
70	1.261	0.263	0.266	0.742	0.177	0.212
70–79	1.270	0.593	0.609	1.211	0.447	0.604
80–89	1.630	0.449	0.076	0.872	0.222	0.591
Above 90	.	.	.	.	.	.
EQ-5D-5L Health states (levels 2–5)						
Mobility	0.956	0.304	0.887	0.886	0.417	0.797
Self-care	0.904	0.582	0.876	0.625	0.377	0.436
Usual activity	0.752	0.281	0.445	0.850	0.288	0.631
Pain/discomfort	1.195	0.299	0.477	1.422	0.444	0.260
Anxiety/depression	0.918	0.277	0.775	0.808	0.181	0.342
Care for family	1.279	0.587	0.592	1.017	0.522	0.973
Care for yourself	1.096	0.209	0.630	1.221	0.284	0.390
cTTO completed	0.532	0.101	0.001	0.371	0.069	0.000

Coefficients represent multiplicative partial effects on the odd ratio of class 2 and class 3 versus class 1 membership

*Coef.* coefficient,* S.E* standard error

Comparing the coefficients of the two scale classes (Table 6) indicated that the effect of both latent pair and matched pair responses was higher on the scale in scale class 2, meaning scale class 2 had a lower error variance (less random class). The effect of task duration among latent pairs was similar between the two scale classes. Compared to the base category (task completed between 15 and 29 s), both shorter and longer times required to complete tasks decreased scale (increased variances). Similarly, for the matched pair, the effect was also similar between the two scale classes; compared to the base category (task completed between 30 and 59 s), shorter duration of tasks increased the scale, and longer duration tasks reduced the scale. The results concluded that the task types induced the major differences in scale classes.

**Fig. 5:**
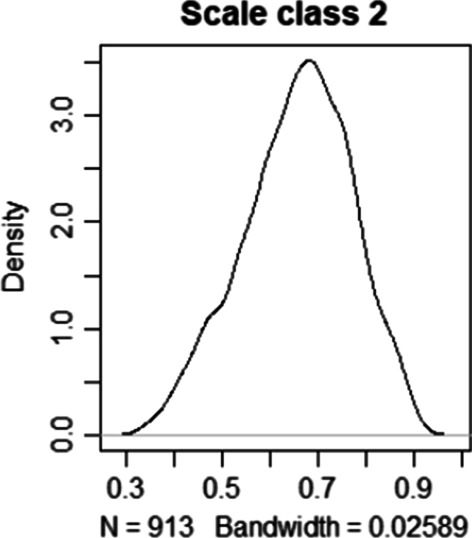
Distribution of Individual grade-of-membership of scale class 2 in 2scale-3taste class SALC model

The inclusion of the hypothesized variables to identify scale classes showed that none of the scale covariates were significant in identifying scale class membership (Table 7). Around 34 percent of the respondents belonged to scale class 1, and around 66 percent belonged to scale class 2. Figure 5 showed the distribution of individual grade-of-membership for scale class 2. Interestingly, respondents of the less random class (i.e., scale class 2) were most likely to be middle and older (45–80 years) who reported a better understanding of health states and were unlikely to complete the cTTO tasks.

## Discussion

In this paper, we explored preference heterogeneity in health valuation among the general Peruvian population. We identified possible task-level factors that might affect the variance within scale classes (heteroskedasticity) and separated latent groups based on different absolute and relative importance (i.e., scale and taste classes). The heteroscedastic model improved the precision of estimated parameters as expected.

The SALC analysis showed that the EQ-5D-5L values in Peru are heterogeneous, with three distinct taste classes. The fundamental difference between the three taste classes was their preference for quality versus quantity of health. All the coefficients on the EQ-5D-5L variables were consistent with its descriptive system in the quality-of-life oriented class (all positive and most of them with significant coefficients). Also, this class's estimated power value for the lifespan attribute was the lowest. In contrast, the power parameter was highest, and the coefficients on the QoL attributes were lowest for the quantity-of-life oriented class. Lastly, a middle class also existed, which fell in between the quality and quantity-oriented classes.

The grade-of-membership results showed a positive association between completing the cTTO and quality-of-life oriented class membership. This suggests that cTTO completion before ordinal tasks caused a paradigm shift in respondents' choices and might influence the burden of health problems. While the SALC analysis can disentangle taste and scale heterogeneity using different sets of covariates, further research is needed to better understand how the cTTO completion influences respondents to select quality over quantity of life (e.g., greater familiarity with health problems and the concept of worse-than-death profiles). In addition, understanding the fundamental nature of people to trade-off between quality and quality of life while valuing their health is an interesting hypothesis that can be further explored in other valuation studies with paired comparison responses only.

A well-known criticism of the SALC model is that the model fails to separate scale and taste heterogeneity [24]. This was addressed in this analysis by incorporating distinct variables in the grade of membership equations (e.g., completion of the cTTO). Overall, the SALC analyses performed well in separating two taste and two scale classes and accounting for heteroskedasticity within the scale classes. However, the small size of this study may have limited the number of classes that could be identified, suggesting further analysis with larger datasets. In conclusion, these latent class analyses provided insight into heterogeneous EQ-5D-5L values in Peru. The study demonstrated taste heterogeneity and its association with scale heterogeneity in the presence of heteroskedasticity. Future studies may extend the SALC analysis under the Zermelo–Bradley–Terry specification similar to the one used in the original study. Furthermore, heterogeneity has been analyzed, assuming that unobserved heterogeneity holds discrete distributions for scale and taste. A hybrid approach may be further explored with continuous distributions. Based on these results, future valuation studies may collect large samples and capture more respondent characteristics and behavioral variables (e.g., experience with health problems) that better identify preference heterogeneity in health valuation.

## Data Availability

The dataset and analysis code are available from the corresponding author upon request.

## Appendix 1

See Figs. 3, 4, 5 and Tables 4, 5, 6 and 7.

**Table 4:** Scale factors of conditional and heteroskedastic logit model

	Coef.	S.E	p-value	Coef.	S.E	p-value
Intercept	0.394	0.024	< 0.001	1.391	0.043	< 0.001
Task type						
Latent pair				.	.	.
Matched pair				− 1.318	0.057	< 0.001
*Duration of each task (s)*						
Latent pair						
1–14				− 0.193	0.080	0.016
15–29				.	.	.
30–59				− 0.320	0.061	< 0.001
Above 60				− 0.308	0.081	0.000
Matched pair						
1–14				0.211	0.076	0.005
15–29				− 0.005	0.052	0.925
30–59				.	.	.
Above 60				− 0.025	0.063	0.693

Coefficients presented in log-scale term. ‘.’ Represented the omitted category

*Coef.* coefficient,* S.E* standard error

**Table 5:** Summary of the converged estimated models

Model	Log-likelihood	Parameters	Bayesian information criterion (BIC)	Akaike information criterion (AIC)	Consistent Akaike information criterion (AIC)
Conditional logit	− 19,377.1	22	38,981.67	38,798.12	39,003.67
Heteroskedastic logit	− 19,041.8	29	38,383.63	38,141.68	38,412.63
Latent class logit (2 classes)	− 17,185.7	81	35,209.13	34,533.34	35,290.13
Scale adjusted latent class logits					
1 Scale and 2 taste classes	− 17,359.2	66	35,400.94	34,850.3	35,466.94
1 Scale and 3 taste classes	− 17,068.9	103	35,203.06	34,343.72	35,306.06
2 Scale and 2 taste classes	− 17,208.5	87	35,316.91	34,591.06	35,403.91
2 Scale and 3 taste classes	− 16,914.7	124	35,112.00	34,077.46	35,236

Models with more classes did not converge

**Table 6:** Heteroskedastic variables of the SALC model

	Scale class 1 (34.21%)			Scale class 2 (65.79%)		
	Coef.	S.E	p-value	Coef.	S.E	p-value
Intercept	0.945	0.072	< 0.001	2.208	0.171	< 0.001
Task type						
Latent pair	.	.	.	.	.	.
Matched pair	− 0.385	0.078	< 0.001	− 0.678	0.189	< 0.001
*Duration of each task (s)*						
Latent pair						
1–14	− 0.223	0.119	0.060	− 0.231	0.278	0.407
15–29	.	.	.	.	.	.
30–59	− 0.357	0.104	0.001	− 0.584	0.214	0.006
Above 60	− 0.389	0.149	0.009	− 0.399	0.252	0.113
Matched pair						
1–14	0.215	0.090	0.017	0.451	0.255	0.076
15–29	0.041	0.059	0.481	0.026	0.109	0.815
30–59	.	.	.	.	.	.
Above 60	− 0.121	0.079	0.123	-0.042	0.099	0.673

Heteroskedastic coefficients presented in log-scale term. ‘.’ Represented the omitted category

*Coef.* coefficient,* S.E* standard error

**Table 7:** Scale class grade-of-membership variables of the SALC model

	Coef.	S.E	p-value
Intercept	0.625	0.302	0.332
Age			
15–29	0.861	0.230	0.576
30–44	–	–	–
45–59	1.308	0.323	0.277
60–80	1.721	0.565	0.098
Gender			
Female	0.775	0.209	0.345
Difficulty level of questionnaire			
Difficult	1.263	0.462	0.524
Understanding of different health state			
Easy/Moderate	1.194	0.317	0.504
Difficulty to choose an answer			
Difficult	0.765	0.186	0.271
cTTO completed	0.529	0.505	0.505
Interview time, excluding cTTO time (min)			
0–10	0.636	0.193	0.136
10–15	–	–	–
Above 15	1.280	0.731	0.666
Interview time—with cTTO (min)			
0–30	0.537	0.766	0.663
30–40	–	–	–
Above 40	0.850	0.559	0.805

Coefficients are in odd ratio

*Coef.* coefficient,* S.E* standard error

## Appendix 2

### Defining the starting values

We have used the standard procedure for conducting the latent class analysis. We conducted a sequence of models, starting with a one-class model and then specifying models with one additional class at a time. Finding the starting values is one of the major issues in implementing the EM algorithm successfully. To make the model insensitive to starting values is important because we seek the global maximum of the log-likelihood rather than a local maximum. As no existing algorithm can distinguish between a global maximum and a local maximum of the log-likelihood, we have repeatedly fit models with randomly selected start values and chosen the solution with the highest converged log-likelihood value. In this study, we did not use any algorithms to select start values for each parameter. Rather, we followed a stepwise method where at the end of the estimation of a given class (K), we used the estimated values of the parameters of the current model as the starting values for the estimation of the additional class (K + 1) model [25]. For the starting value of the dependent variable parameters of the additional class, we started with random variables from a uniform distribution range from 0 to 1. Also, for the starting values of the covariate parameters of the additional class, we used starting values of 0 followed by the Latent Gold software package [26].

## Abbreviations

BWS: Best–worst scaling
EQ-5D-5L: EuroQol 5 domains 5 levels
EQ-VT: EuroQol valuation technology
GOM: Grade-of-membership
HPR: Health preference research
HRQoL: Health-related quality of life
QALY: Quality-adjusted life-year
QoL: Quality of life
SALC: Scale-adjusted latent class
cTTO: Composite time trade-off

## Footnotes

1
$Q$ represents the quality of life and $T$ represents length of life.

2 EQ-5D-5L has five attributes: MO = mobility, SC = self-care, UA = usual activity, AD = anxiety/depression, PD = pain/discomfort.

3 On a QALY scale, the coefficient of the first level (1) of each dimension is 0 by construction ${V}_{1}\left(11111\right)=1$, for which the regression has 20 parameters.

4 In the probability function, ${y}_{itj}$ represents a dummy (binary) variable, taking a value 1 if individual $i$ has chosen health profile $j$ in task $t$, and a value 0 otherwise.

5 The scale function is in log scale. So, the effect of the latent pair is exp (1.3914) = 4.020, and the effect of matched pair is exp (1.3914–1.3176) = 1.077.
